# Formation of silicon nanostructures with a combination of spacer technology and deep reactive ion etching

**DOI:** 10.1186/1556-276X-7-288

**Published:** 2012-06-06

**Authors:** Daniel CS Bien, Hing Wah Lee, Siti Aishah Mohamad Badaruddin

**Affiliations:** 1 Nanoelectronics Cluster, MIMOS Berhad, Technology Park Malaysia, Kuala Lumpur 57000, Malaysia

**Keywords:** Silicon, Nanostructures, Nano-masking, High-aspect ratio, Deep reactive ion etching, Spacers

## Abstract

A new method of fabricating high aspect ratio nanostructures in silicon without the use of sub-micron lithographic technique is reported. The proposed method comprises two important steps including the use of CMOS spacer technique to form silicon nitride nanostructure masking followed by deep reactive ion etching (DRIE) of the silicon substrate to form the final silicon nanostructures. Silicon dioxide is used as the sacrificial layer to form the silicon nitride nanostructures. With DRIE a high etch selectivity of 50:1 between silicon and silicon nitride was achieved. The use of the spacer technique is particularly advantageous where self-aligned nanostructures with potentially unlimited lengths are formed without the need of submicron lithographic tools and resist materials. With this method, uniform arrays of 100 nm silicon nanostructures which are at least 4 μm tall with aspect ratio higher than 40 were successfully fabricated.

## Background

As microdevices shrinks towards nanoscale, formation of high aspect ratio nanostructures will be more challenging. These nanostructures has numerous applications such as photonic crystals [1,2], thermoelectric generators [3], sensors [4], resonators [5], nanocapacitors [6] and nano-molds [7] for nanoimprint lithography. The aspect ratio of the device is defined by the depth to width ratio of the structure.

Typically, in semiconductor device fabrication, a combination of sub-micron lithography techniques and etching are commonly used in generating patterns with nano dimensions. Such techniques includes electron beam lithography [8], dip-pen lithography [9], near field scanning probe lithography [10], nanoimprint lithography [7] and x-ray lithography [11]. However, these techniques might not be suitable to produce high aspect ratio nanostructures as there is resist imposed limitations during etching, namely the resist thickness is thin and unable to withstand long durations of high power plasma etching. Alternatively, silicon nanostructures or nanowires can also be synthesized by bottoms-up method via chemical vapour deposition [12], laser-ablation [13] and thermal evaporation [14] techniques. However, organising these nanowires into ordered arrays is challenging and the synthesis process often requires the use of metal catalyst or nano-powders which are not compatible with the standard CMOS fabrication processes.

In this letter, we demonstrate a new method of forming high aspect ratio silicon nanostructures, where very accurate alignment of the nanostructures can be achieved because the alignment is not determined by the lithographic tool but by the spacer technique used. The fabrication method is divided into two parts where arrays of silicon nitride nanostructures are first formed by the CMOS spacer method which is typically used in the fabrication of nanometer transistors. The formed nitride nanostructured arrays are then used as a masking layer during the silicon etching process. To produce an array of silicon nanostructures, we etch the silicon substrate in an inductively coupled plasma, Tegal AMS 110 DRIE system. Further details are described in the following methodology and results sections.

## Method

**Figure 1:**
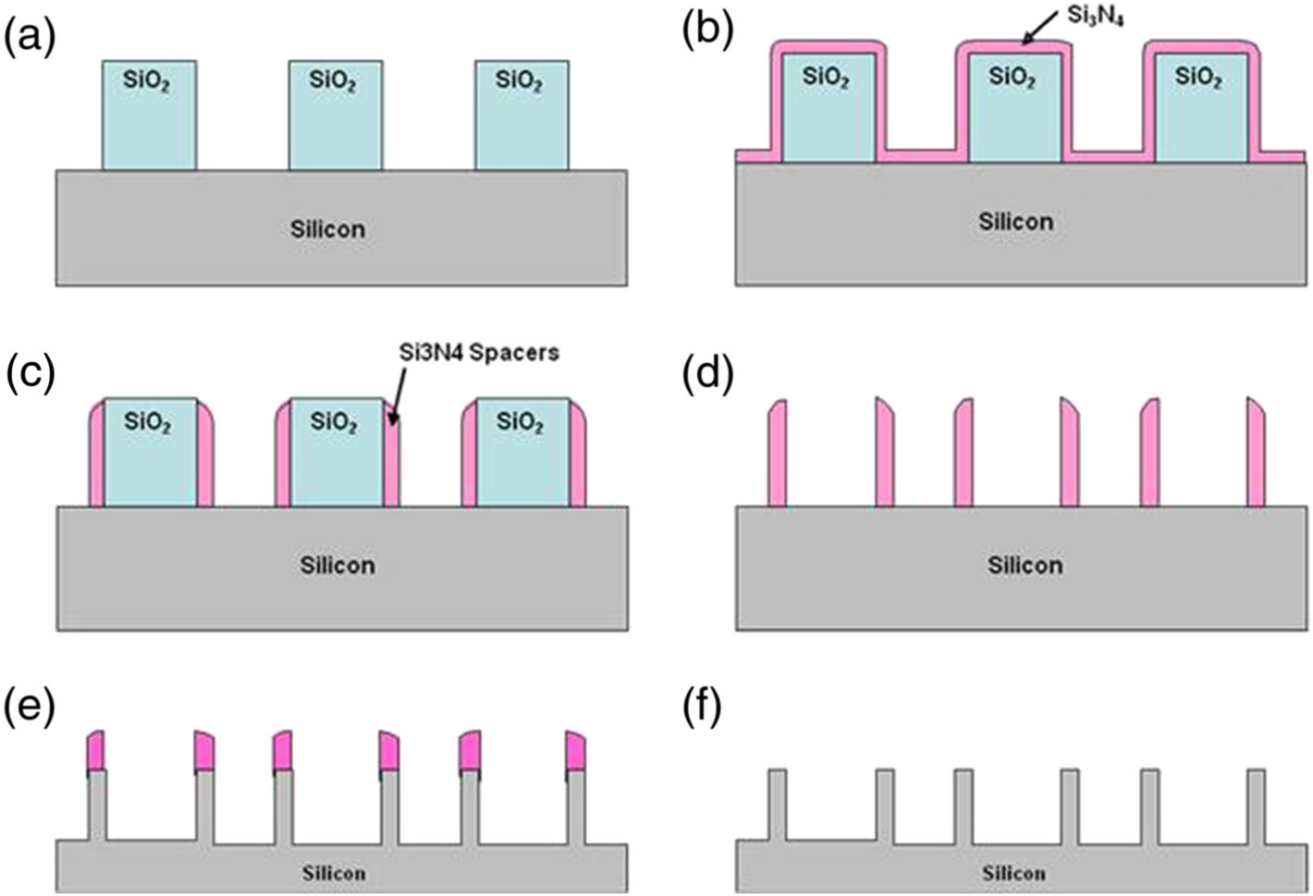
Process flow for fabricating silicon nanostructures with a combination of silicon nitride nano-spacers and deep reactive ion etching of silicon.

The process of forming the silicon nanostructures is illustrated in Figure 1. All experiments were conducted on 700 μm thick, 200 mm diameter silicon substrates. First, a 200 nm thick silicon dioxide (SiO2) layer was thermally grown by wet oxidation. The SiO_2_ layer was then photolithographically patterned into lines and etched with a combination of CF_4_ and CHF_3_ plasma in a reactive ion etching system (Figure 1a). A silicon nitride (Si_3_N_4_) layer is deposited onto the SiO_2_/Silicon surfaces by low pressure chemical vapor deposition, LPCVD (Figure 1b). Silicon nitride spacers were then formed by time controlled etching of the nitride layer with tetrafluoromethane (CF_4_) and trifluoromethane (CHF_3_) plasma (Figure 1c). The silicon nitride spacers will be used as an etch mask during the formation of the silicon nanostructures. The widths of the nitride spacers are in correlation with the deposited thickness of the PECVD nitride layer and are also dependent on the directionality of the plasma etch. The SiO_2_ layer was then selectively removed in a buffered hydrofluoric acid (HF) solution leaving behind an array of silicon nitride spacers or nanostructures (Figure 1d). When characterizing the etch rates of oxide and nitride in the buffered HF solution it was observed that oxide was etched at a rate of 70 nm/min while the etch rate of nitride in the same solution was approximately 2 nm/min, showing very high selectivity.

To form the high aspect ratio silicon nanostructures, the silicon substrate with nitride nano-masking was etched in an inductively coupled plasma (ICP) deep reactive ion etching system (Figure 1e). Etching of the silicon utilizes an alternating sulfur hexafluoride (SF_6_) and octafluorocyclobutane (C_4_F_8_) plasmas, where SF_6_ is used as the etch gas and C_4_F_8_ as the passivation gas. During the etch process, the substrate was mechanically clamped and cooled by helium backside pressure to maintains a low temperature at the substrate surface. Detailed process conditions are shown in Table 1. Finally the silicon nitride nano-mask can be removed leaving behind only the silicon nanostructures (Figure 1f) by etching in buffered HF or in an orthophosphoric acid solution with an etch rate of 20 nm/min at 175°C. The fabricated structures were characterized with a JEOL scanning electron microscope (SEM).

**Table 1:** **DRIE of silicon with SF**_**6**_**and C**_**4**_**F**_**8**_**plasma**

**Process Conditions**	**Value**
Source Power	1500 W
Bias Power	12 W
SF_6_ Flow	250 sccm
C_4_F_8_ Flow	300 sccm
Silicon Etch Rate	1.5 μm/min

## Results and discussions

**Figure 2:**
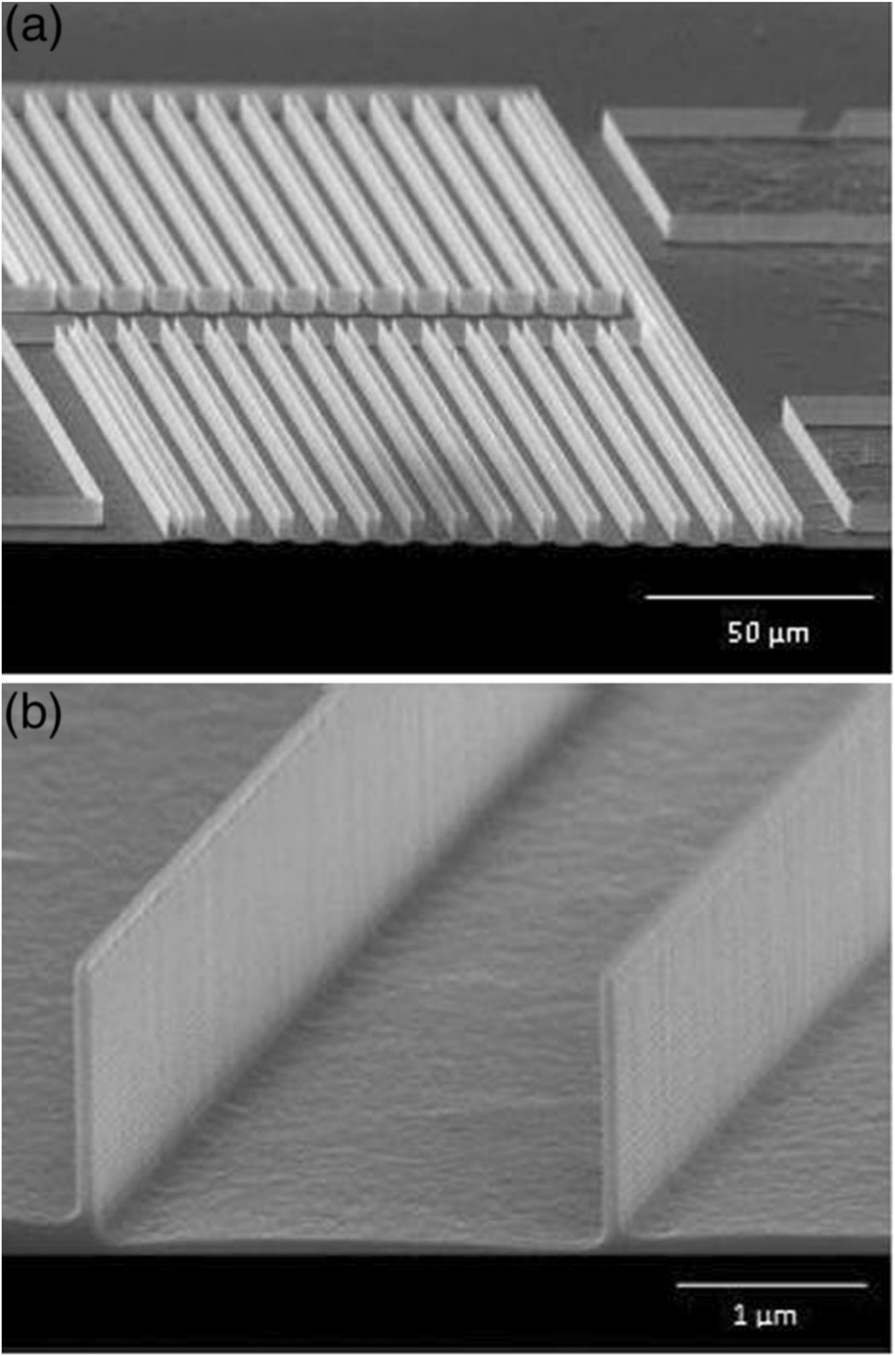
SEM images of (a) an array; and (b) a close-up view of silicon nanostructures formed with a combination of spacer method and deep reactive ion etching.

Utilising a combination of spacer method and deep reactive ion etching presented in the previous section, fabricated silicon nanostructures is illustrated in Figure 2, showing an array (Figure 2a) and a close-up view (Figure 2b) of the formed nanostructures. Results achieved shows good etch selectivity between silicon and silicon nitride (Si_3_N_4_) with silicon etch rate of 1.5 μm/min and an etch selectivity value of 50:1 between silicon and Si_3_N_4_. A higher etch selectivity of 70:1 was observed between silicon and silicon dioxide (SiO_2_). However, in this work SiO_2_ is used as the sacrificial layer to form the Si_3_N_4_ nano-masking. It is desirable that at least 20 nm of the mask layer remains after etching to inhibit roughening of the silicon surface.

**Figure 3:**
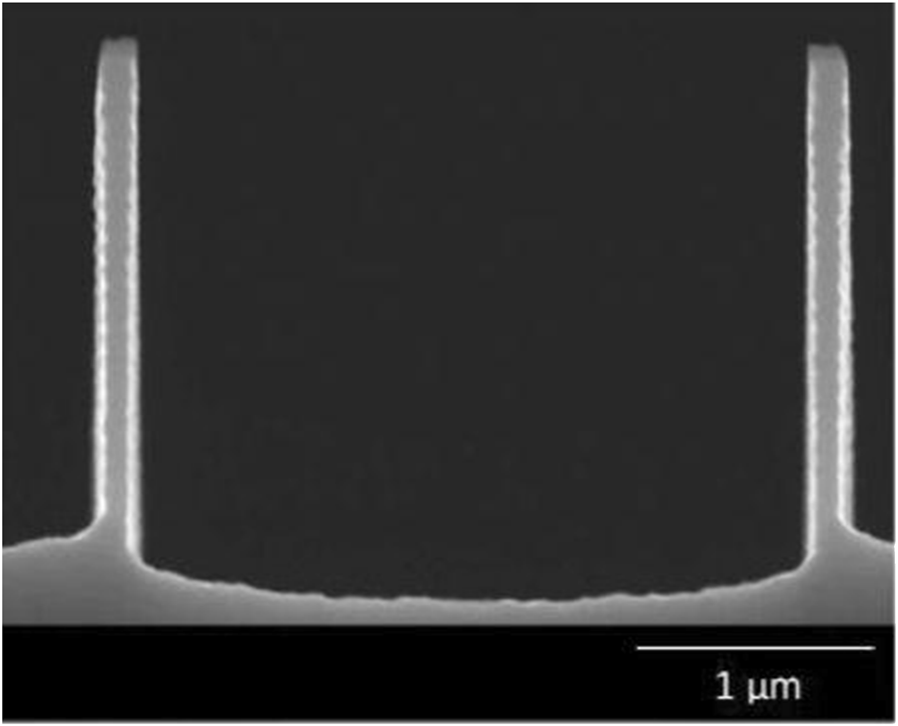
Fabricated 100 nm silicon nanostructures with at least 20:1 aspect ratio.

**Figure 4:**
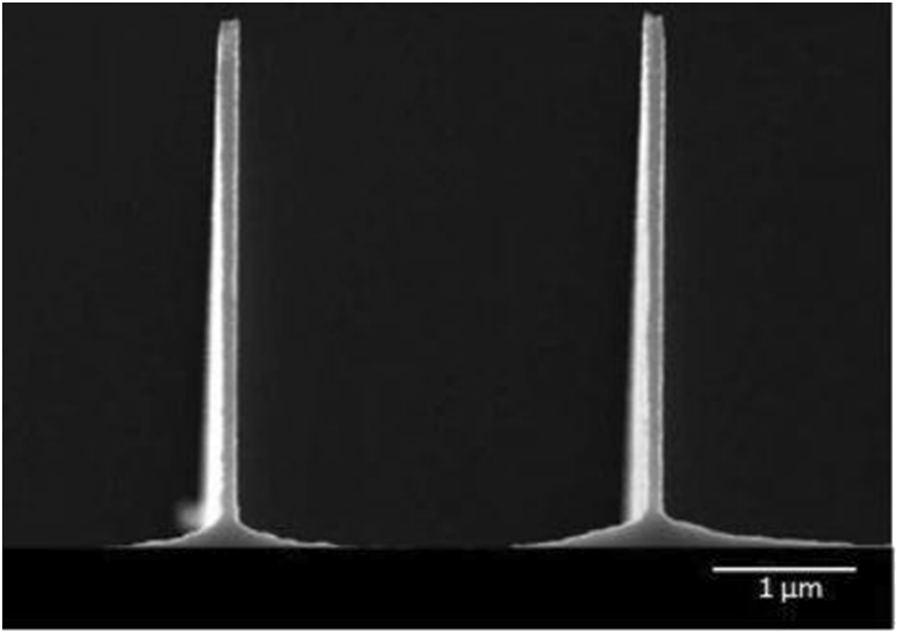
Fabricated 100 nm silicon nanostructures with at least 40:1 aspect ratio.

Significantly high aspect ratio silicon nanostructures were successfully formed with this method. 100 nm nanostructures were etched into the silicon substrate to a depth of 2.3 μm and 4.2 with aspect ratios of 23 and 42 as illustrated in Figures 3 and 4 respectively. The achieved aspect ratio of at least 40 is much higher than those previously published by researchers working with nanostructures. An aspect ratio of 20 was achieved by Chang et al. [15] with a combination of nickel masking and reactive ion etching; aspect ratio of 4.4 achieved by Suh et al. [16] by nanoimprinting and capillary force lithography; aspect ratio of 5.5 achieved by Cho et al. [17] by nanoimprinting with poly-mthyl-methacrylate (PMMA); aspect ratio of 3 achieved by Kwon et al. [10] using near field scanning optical lithography and potassium hydroxide etching with silicon nitride masking; aspect ratio of 25 achieved by Henry et al. [18] by cryogenic silicon etching with alumina masking; aspect ratio of 10 achieved by Peroz et al. [19] by step and repeat nanoimprint lithography; and aspect ratio of 10 achieved by Gowrishankar et al. [20] by block copolymer lithography and NF_3_ based reactive ion etching.

Besides having high aspect ratio nanostructures, very accurate structure alignment are achieved because the structure alignment is not determined by lithographic tool but by the spacer method, where the length of the fabricated structures can be unlimited. With the spacer method, dimensions as fine as 10 nm can be achieved. However, there is a dimension limitation during the silicon etch with SF_6_ and C_4_F_8_ plasma as the etch process would typically create sidewall scalloping greater than 10 nm. Further etching of silicon nitride in buffered HF can further reduce the width of the nanostructures, where the lower limit of the achievable width of the nanostructures is dependent on the silicon dioxide thickness, silicon nitride thickness, uniformity of the nitride coverage and directionality of the etch process. It is also possible to reduce the dimensions of the nanostructures through lateral isotropic plasma etching of the nitride spacers where the process can be controlled by varying the etch recipes used which is dependent on gas ratios, chamber pressure and rf power. However, to achieve accurate directional control is difficult and the etch rate of nitride can still be high. With wet etching, the etch rate of nitride in buffered HF was found to be only 2 nm/min which allows better control of the final nitride dimensions.

## Conclusions

In summary, we have demonstrated a method of fabricating high aspect ratio nanostructures in silicon using a combination of CMOS spacer method to form silicon nitride nanostructure masking and deep reactive ion etching of silicon with SF_6_ and C_4_F_8_ plasma for applications in photonics, photovoltaic and nano-electromechanical (NEM) devices. The demonstrated fabrication method is cost effective where it does not require the use of sub-micron lithographic tools and techniques. Alignment of the silicon nitride nano-masking can be controlled accurately and the final silicon nanostructures formed are of aspect ratio higher than 40 which is significantly higher than that produced previously for nanostructures. In this work, etch selectivity between silicon and silicon nitride of approximately 50:1 was achieved and the authors believe that the dimensions of the nanostructures can be further reduced by thinning the silicon nitride nano-masking in buffered hydrofluoric acid solution.

## Competing interests

The authors declare that they have no competing interest.

## Authors’ contributions

DCS Bien coordinated the research work and conceived the fabrication process flow. HW Lee participated in conceiving the process flow and conducted characterization of the nanostructures and SA MB fabricated the nanostructures. All authors read and approved the final manuscript.
